# A cutaneous metastasis of unresectable rectal adenocarcinoma: A case report and literature review

**DOI:** 10.1016/j.ijscr.2020.04.102

**Published:** 2020-05-19

**Authors:** Riyadh Hakami, Mohammed N. Alali, Turki Alshammari, Sulaiman AlShammari, Zyad Alyahya, Mohammed Ayesh, Khaled AlSaad, Alaa Abduljabbar

**Affiliations:** a Department of Surgery, College of Medicine, King Saud University Medical City, King Khalid University Hospital, Riyadh, Saudi Arabia; b Department of Surgery, King Faisal Specialist Hospital and Research Center, Riyadh, Saudi Arabia; c Department of Pathology and Laboratory Medicine, King Faisal Specialist Hospital and Research Center, Riyadh, Saudi Arabia; d Department of Radiology and Medical Imaging, College of Medicine, King Saud University Medical City, Riyadh, Saudi Arabia

**Keywords:** Rectal adenocarcinoma, Colorectal cutaneous metastases, Groin skin metastasis, Cutaneous cancer, Cutaneous nodule

## Abstract

**Background:**

Colorectal cancer is ranked third among the most commonly diagnosed malignancies and fourth among the leading causes of cancer death in the world. However, only a few case reports are found in the literature regarding skin metastasis originating from rectal cancer, which usually shows widespread disease and poor prognosis. Approximately, 0.8% of the patients will have skin lesion as the first indication of a silent internal malignancy, which is rare.

**Case report:**

We report a complicated case of a 45-year-old male patient who referred to our highly specialized governmental hospital for diversion loop colostomy as well as biopsies of rectal and inguinal skin areas followed by palliative radiation therapy to the pelvis. Histopathological exam of rectal biopsies revealed moderately differentiated rectal adenocarcinoma, while the skin of the right inguinal area showed metastatic cutaneous rectal adenocarcinoma. Unfortunately, palliative radiation therapy was not started as the patient passed away secondary to respiratory failure which ended by cardiopulmonary arrest.

**Conclusion:**

A patient who is having new or evolving skin lesions with an oncology history should be well investigated as cutaneous metastasis is a strong possibility.

## Highlights

• Colorectal cancer (CRC) is the third most commonly diagnosed malignancy and the fourth leading cause of cancer death in the world.
• Approximately, 0.8% of patients will present with skin lesion as the first sign of a silent internal malignancy as skin metastasis without visceral metastasis is rare in CRC.
• A comprehensive literature review (including clinical features of patients, management, and outcome) covering all reported cases of cutaneous metastasis secondary to rectal cancer was included for better understanding of the disease.

## 1 Introduction

The concerns regarding increased colorectal cancer (CRC) incidence and mortality are increased in many medium-to-high human development index (HDI) regions including Eastern Europe, Asia, and South America compared to the highest indexed HDI regions including USA, Australia, New Zealand, and several Western European countries [1]. Currently, CRC is ranked third among the most commonly diagnosed malignancies and fourth among the leading causes of cancer death in the world. By 2030, the estimate increase in the burden of CRC is about 60% denoting more than 2 million newly diagnosed patients and 1.1 million cancer-caused deaths [2]. Metastatic skin cancer occurs from internal malignancy, which is considered extremely rare, representing only 0.001% of all skin biopsies performed [3].

Skin cancer related to CRC accounts only for about 6.5% of all biopsied lesions, most often metastasize to liver and lung. Approximately, 0.8% of patients will have skin lesion as the first indication of a silent internal malignancy which is rare compared to metastasis that occurs a few years after the detection or resection of the primary tumor (usually developing within the first 3 years of follow-up). Spared skin metastasis without visceral metastasis is rare in CRC [[3], [4], [5], [6]]. This project has been reported in line with the SCARE criteria [46].

We are reporting a complicated case of locally advanced low rectal cancer with extensive metastasis to inguinal and perineal skin and distant metastasis to multiple organs in a middle-aged Saudi male patient who was treated as a palliative case. A comprehensive literature review (including clinical features of patients, management, and outcome) covering all reported cases of secondary to rectal cancer was included for better understanding of the disease.

## 2 Case report

**Fig. 1:**
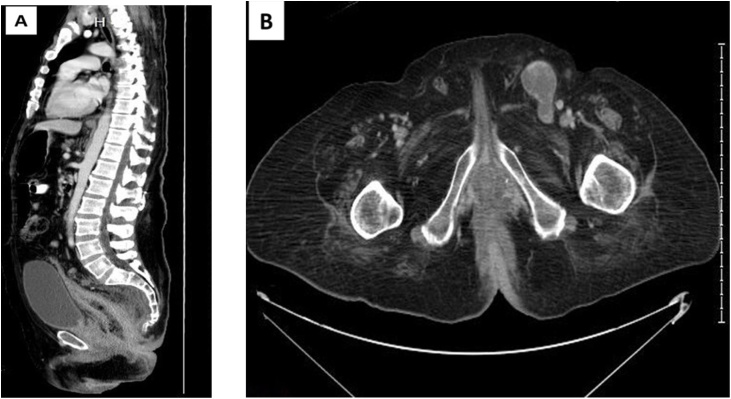
Sagittal and axial (B) reformatting for abdomen and pelvis CT scan show diffuse wall thickening of the colon with haziness of the meso-rectal fat planes and multiple regional enlarged lymph nodes. The thickening is extending into the anus. There are also multiple enlarged lymph nodes in inguinal areas.

**Fig. 2:**
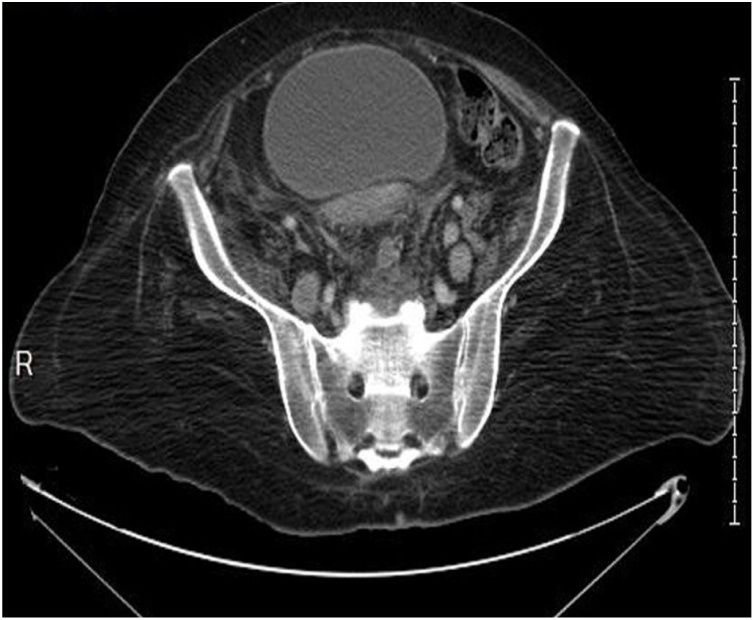
Axial CT scan of the pelvis shows multiple enlarged lymph nodes seen along the iliac vessels.

**Fig. 3:**
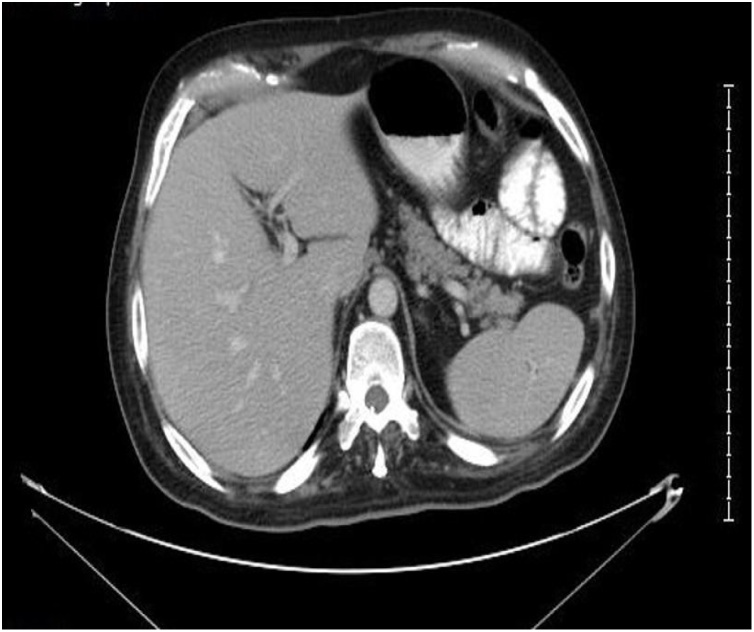
CT scan upper abdomen with IV contrast shows no abdominal organs metastases.

A 45-year-old male patient, known to have white matter leukodystrophy and generalized spasticity of unknown etiology which started at the age of 14, was progressive and kept bedridden for the past 7 years for which a Baclofen pump was inserted. Also, he had mental disability. The patient was diagnosed with locally advanced low rectal cancer with distant metastasis to multiple organs including perineal and inguinal skin, lung, external iliac, colon, and inguinal lymph nodes metastasis. In 2018, about 1 month prior to referral to King Faisal Specialist Hospital and Research Center, the patient was following with Neurology regarding his condition, where he was found to have lower GI bleeding and surgery was involved. He underwent investigation for lower GI bleeding including colonoscopy, and he was found to have rectal mass and could not pass the scope above it as well as the skin lesion which was biopsied. The case was discussed in the multidisciplinary tumor board and planned for diversion loop colostomy as well as rectal biopsies and inguinal area skin biopsy followed by palliative radiation therapy to the pelvis. Computed Tomography (CT) scan of the abdomen and pelvis (Fig. 1, Fig. 2, Fig. 3) demonstrated a circumferential enhancing wall thickening involving the whole rectum with ill-defined hypodense area seen 7–9 o’clock with possible involvement of the anal canal associated with diffuse edema and fat stranding of the mesorectum. There were multiple necrotic lymph nodes noted in the mesorectum and bilateral internal iliac region, the largest one in the right internal ilium measuring 2 cm. There were multiple necrotic lymph nodes seen on the right external iliac (measuring 1.6 cm) and bilateral inguinal area, the largest one on the left side measuring 3.4 × 3.3 cm. There were bilateral symmetrical hilar necrotic lymph nodes measuring on the right side 2.5 × 2 cm and on the left side 3.2 × 1.6 cm. At the perivascular space, they measured 1 cm, being at least T3 N2.

**Fig. 4:**
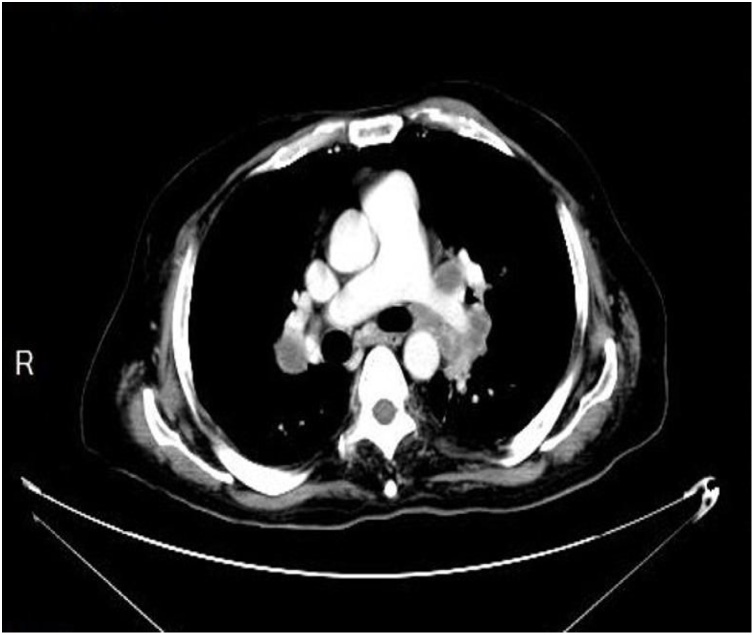
CT scan of the chest with IV contrast shows bilateral hilar and mediastinal enlarged lymph nodes.

**Fig. 5:**
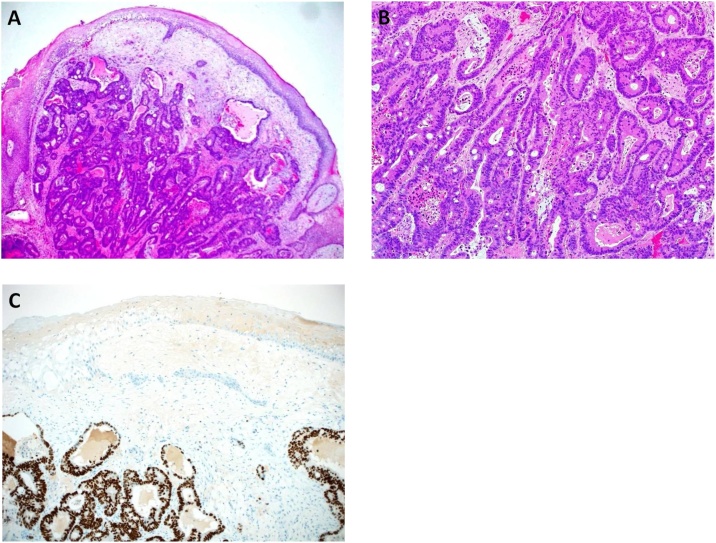
a) The tumor involved the dermis and subcutaneous tissue [hematoxylin-eosin (H&E), original magnification ×40]. b) The tumor consisted of complex and single neoplastic glandular structures with intervening desmoplastic stroma [H&E, original magnification ×100]. c) Immunohistochemical staining for CDX2 shows diffuse and strong nuclear staining in the neoplastic cells [original magnification ×100].

Magnetic Resonance Imaging (MRI) for local staging was contraindicated as the patient was with an implanted pump. CT Chest (Fig. 4) demonstrated multiple bilateral tiny pulmonary nodules, the largest one measuring 4 mm in the right upper lobe. Upon examination, the patient was bedridden with poor functional status. Glasgow coma score (GCS) was 15/15, having generalized spasticity. Perineal examination revealed multiple exophytic masses in the scrotal skin, inguinal folds, and perineum and gluteal folds, which were firm to hard in consistency with a few being ulcerated especially over scrotum with little oozing of the serosanguinous fluid. There was no mechanical obstruction of orifices (anal or urethral). The patient was in the general surgical ward to continue postoperative care and management. Histopathological exam of rectal biopsies revealed moderately differentiated rectal adenocarcinoma, while the skin of the right inguinal area showed metastatic cutaneous rectal adenocarcinoma (Fig. 5a, b, c). Unfortunately, later, the patient developed respiratory failure secondary to aspiration pneumonia which ended by cardiopulmonary arrest and death.

## 3 Discussion

CRC is the most common cancer affecting males and the third common cancer affecting females in Saudi Arabia, which accounts for 11.5% of the newly diagnosed cases in 2014 [39]. Krathen et al. conducted a meta-analysis which demonstrated the incidence of colorectal cutaneous metastasis as ranging between 3.4% and 4.0% [4,40].

Worldwide, a few case reports were reported regarding skin metastasis associated with rectal cancer, and none of them was from the Kingdom of Saudi Arabia or Arabic Gulf countries. Accordingly, we report the first case report of palliative Saudi male patient diagnosed with rectal cancer associated with extensive skin metastasis to the perineum and inguinal area.

Grossly, the specimen consisted of a single, dome-shape piece of skin with the underlying soft tissue, measuring 0.8 × 0.6 × 0.3 cm. The skin was intact and showed no evidence of ulceration. Sections from the tumor were fixed in 10% buffered formalin, paraffin-embedded, sectioned at 5 μm, mounted on coated glass-slides, and stained by routine hematoxylin and eosin stain. A CDX2 immunohistochemical (IHC) stain (clone EPR2764Y, Ventana) with proper positive control study was performed following the manufacturer’s guidelines, using an automated platform (Ventana Benchmark XT, Tucson, AZ, USA), and heat antigen retrieval by ultracell condition solution PH 8.4. An ultra-view universal DAB detection kit was used for reaction visualization.

Histopathological examination showed polypoid, moderately differentiated adenocarcinoma, which involved the dermis and subcutaneous soft tissue (Fig. 5a). The tumor consisted of complex and single glandular structures with intervening desmoplastic stroma. The neoplastic cells exhibited a moderate degree of pleomorphism and had round, hyperchromatic and vesicular nuclei, visible nucleoli, moderate amount of cytoplasm, and poorly-defined cell membranes (Fig. 5b). Mitoses, single cell necrosis, and central, comedo-type necrosis were seen. The neoplastic cells exhibited diffuse and strong nuclear positivity for CDX2 (Fig. 5c). The overlying epidermis showed mild hyperkeratosis with no dysplasia or melanocytic atypia. The pathological findings showed compatability with colorectal metastatic adenocarcinoma.

Skin metastasis usually developed during the first three years postoperatively or the diagnosis of rectal cancer which may be the first sign or symptom of asymptomatic, unsuspected occult malignancy associated with poor prognosis, a failure of ongoing management or recurrence. Multiple theories of the possible rout of skin metastasis were introduced. These included direct extension, lymphatics, and implantation at the time of biopsy or operative procedure, hematogenous spread, and spread along the embryonic ligaments [4,28].

CRC metastases to the skin are well-differentiated, often mucin-secreting adenocarcinomas. They are typically presented with a rapidly growing painless flesh-colored dermal or subcutaneous nodule or as a mass with occasional ulceration. Cutaneous metastasis is mostly on the postoperative scars. It is also found, however, on the extremities, head, neck, and penis skin. It commonly denotes widespread disease and poor prognosis with an estimated median survival of 18–20 months after the occurrence of skin metastases [[3], [4], [5]].

An extensive literature review was conducted which demonstrated a limited number of reported cases demonstrated as the following: Reingold investigated 2300 cases of internal malignancies through autopsy studies and found only one case of skin metastasis secondary to rectal cancer out of 32 cases of cutaneous metastases [4,41]. At Hershey Medical Center in Pennsylvania, two wide-scale retrospective studies were conducted by Lookingbill et al. investigating the cutaneous metastases of internal malignancies as they had four and eighteen cases, respectively, but the result did not clearly show whether the metastasis is secondary to colon or rectum cancers [28,42].

Up to date, only 43 cases of cutaneous metastases secondary to rectal cancer were reported, as 28 cases were found by Dehal et al. as listed in Table 1, in addition to 15 cases found by Hakami et al. as listed in Table 2. The patients had a mean age of 59 years (range 29–84), with male predominance as 27 were men; the majority of them presented with skin nodules and had adenocarcinoma as the underlying histology. High-risk features such as mucinous (n = 8), signet ring cell (n = 4), and poor differentiation (n = 7) appeared in 44.18% of the patients. In our case, histopathological exam of the skin of right inguinal area revealed skin with cutaneous adenocarcinoma, consistent with metastatic rectal adenocarcinoma, which consists of a single irregular piece of pale tan soft tissue measuring 0.8 × 0.6 × 0.3 cm. However, the rectal biopsies consisted of multiple tiny pieces of cream-tan soft irregular tissue measuring in aggregate 0.7 × 0.3 × 0.2 cm which revealed moderately differentiated rectal adenocarcinoma. Immunohistochemical stains were done as the assayed sample carried the mutation c.34G>T (p.G12C) in exon 2 of the *K-RAS* oncogene. No mutations were found in exons 2, 3, and 4 of the *N-RAS* oncogenes and in exons 11 and 15 of the *BRAF* gene.

**Table 1:** Cases of rectal cancer with cutaneous metastasis (Dehal et al.).

Author, year	Age, years	Sex	Histology	Stage	Primary cancer treatment	Interval,^a^ months	Skin mets location	Skin mets morphology	Skin mets treatment	Survival (follow-up time in months)
Gray and Das, [7] 1989	79	F	Adenocarcinoma	–	Radiation	0^b^	Leg	Nodules	None	No (18)
Reed and Stoddard, [8] 1992	68	F	Adenocarcinoma, poorly differentiated	–	LAR	4	Perineum	Nodules	APR	–
De Friend et al., [9] 1992	49	F	Adenocarcinoma	III	LAR	7	Perineum	Nodules	WLE	–
Kauffman and Sina, [10] 1997	50	M	Adenocarcinoma, signet ring	IV	LAR + ACR	36	Multiple	Plaques	None	No (3)
Adani et al., [11] 2001	70	F	Adenocarcinoma	III	APR + AC	36	Leg	Nodules	CR	Yes (14)
Tsai et al., [12] 2002	47	M	Adenocarcinoma, signet ring	III	APR + AC	11	Multiple	Nodules	C	No (4)
Melis et al., [13] 2002	41	M	Adenocarcinoma	IV	NCR	1	Perineum	Plaques	None	–
Damin et al., [14] 2003	44	M	Adenocarcinoma	II	LAR	6	Groin	Zosteriform	R	No (5)
Hayashi et al., [15] 2003	50	M	Adenocarcinoma, mucinous	–	LAR	4	Perineum	Nodules	None	–
Sarid et al., [16] 2004	60	F	Adenocarcinoma, mucinous	III	NR + LAR + ACR	16	Chest, abdomen	Ulcers	WLE	No (56)
Reuter et al., [17] 2006	69	M	Adenocarcinoma	II	APR + ACR	5	Perineum	Plaques	None	No (6)
Tan et al., [18] 2006	70	M	Adenocarcinoma, mucinous	IIIb	LAR + AC	24	Back	Nodules	WLE, C	–
Tan et al., [18] 2006	53	F	Adenocarcinoma	IIIb	APR	10	Perineum	Nodules	WLE, CR	No (26)
Kilickap et al., [19] 2006	29	M	Adenocarcinoma, signet ring	IIIa	LAR + APR + ACR	14	Chest wall, axilla	Nodules	WLE, C	Yes (4)
Gazoni et al., [20] 2008	55	F	Adenocarcinoma, poorly differentiated	IV	Colostomy + CR	0^b^	Perineum	–	CR	No (3)
Gazoni et al., [20] 2008	66	M	Adenocarcinoma, poorly differentiated	IV	Colostomy + CR	0^b^	Perineum	–	CR	No (4)
Gazoni et al., [20] 2008	68	M	Adenocarcinoma, poorly differentiated	IV	Colostomy + CR	0^b^	Thigh, axilla	–	CR	No (3)
Gazoni et al., [20] 2008	72	M	Adenocarcinoma	IV	Colostomy + CR	0^b^	Perineum	–	CR	No (5)
Gazoni et al., [20] 2008	65	M	Adenocarcinoma	IV	Colostomy + CR	0^b^	Perineum	–	CR	No (7)
Gazoni et al., [20] 2008	78	M	Adenocarcinoma	IV	Stent + CR	0^b^	Perineum	–	CR	No (1)
McWeeney et al., [21] 2009	72	M	Adenocarcinoma	III	Ileostomy + NCR	6	Perineum	Nodules	WLE	–
Saladzinskas et al., [22] 2010	64	M	Adenocarcinoma, mucinous	IIa	NR + LAR	42	Face	Ulcers	WLE	Yes (7)
Ismaili et al., [23] 2011	50	F	Adenocarcinoma, signet ring	IV	None	0^b^	Multiple	Zosteriform	None	No (1)
Balta et al., [24] 2012	46	M	Adenocarcinoma, mucinous	IIIb	Colostomy	12	Perineum	Ulcers	None	–
de Miguel Valencia et al., [25] 2013	55	M	Adenocarcinoma, mucinous	IIIb	NCR + APR + AC	18	Multiple	Nodules	None	No (—)
Ozgen et al., [26] 2013	65	M	Adenocarcinoma	IIa	NCR + LAR + ACR	18	Perineum	Nodules	CR	Yes (12)
Akpak et al., [27] 2013	47	F	Adenocarcinoma	IV	APR	36	Perineum	Ulcers	WLE + CR	–
Dehal et al., [28] 2015	47	M	Adenocarcinoma	IV	CR	1	Perineum	Nodules	R	Yes (12)

– = data not reported; AC = adjuvant chemotherapy; ACR = adjuvant chemoradiation; APR = abdominoperineal resection; C = chemotherapy; CR = chemoradiation; F = female; LAR = low anterior resection; M = male; mets = metastasis; NCR = neoadjuvant chemoradiation; NR = neoadjuvant radiation; R = radiation; WLE = wide local excision.

a Interval between cancer treatment/diagnosis and skin metastasis presentation.

b In those patients, skin metastasis was the first sign of the underlying malignancy. Therefore, there was no interval between the primary cancer diagnosis and the onset of the skin metastasis.

**Table 2:** Additional cases of rectal cancer with cutaneous metastasis (current study).

Author, year	Age, years	Sex	Histology	Stage	Primary cancer treatment	Interval,^a^ months	Skin mets location	Skin mets morphology	Skin mets treatment	Survival (follow-up time in months)
Nasti G et al., [29] 2007	76	F	Adenocarcinoma	IIIb	NCR	0^a^	Parotid skin and Frontal face	Nodules	None	No (15)
Tranchart et al., [30] 2008	59	F	Adenocarcinoma, well differentiated	IIa	LP + TME + ISR	14^a^	Perianal	Nodules	WLE + C	No (16)
Tranchart et al., [30] 2008	70	M	Adenocarcinoma, well differentiated	IIa	NCR + P + TME	10^a^	Perianal	Nodules	WLE	Yes (22)
Goris et al., [31] 2011	79	M	Adenocarcinoma	–	Resection (-)	36^a^	Pubis, penis and scrotum	Nodule	None	No (6)
Balta et al., [32] 2013	84	F	Adenocarcinoma	IV	NCR	–	Occipital	Nodule	NCR	Yes (-)
Miguel Valencia et al., [33] 2013	55	M	Adenocarcinoma, well differentiated, mucinous	III	NCR + APR	–	Pectoral	Nodules	No	No (-)
Kitahara et al., [34] 2014	52	M	Adenocarcinoma, moderately differentiated	IIc	CR + TPE	–	Perineum	Nodules	Extended TPE	No (36)
Kitahara et al., [34] 2014	38	M	Adenocarcinoma, moderately differentiated	IV	CR + TPE	–	Perineum	Nodules	Extended TPE	Yes (60)
Kitahara et al., [34] 2014	50	F	Adenocarcinoma, poorly differentiated	IIc	CR + APR	–	Perineum	Nodules	APR	Yes (24)
Yazilitas et al., [35] 2015	50	F	Adenocarcinoma	–	NC + resection + ACR	7^a^	Forehead	Nodule	None	-(24)
Liasis, L. et al., [36] 2016	61	M	Adenocarcinoma, poorly differentiated	IIa	NCR + APR + ACR	2^a^	Perineum	Ulcer	APR	Yes (60)
Wang et al., [37] 2017	76	F	Adenocarcinoma, poorly differentiated	IIIc	LAR	0^b^	Back, Gingiva	Nodules	None	No (3)
Hamid et al., [38] 2017	75	F	Adenocarcinoma, well differentiated	–	NCR + P + TME	14^a^	Perianal	Nodules	None	–
Yagnik et al., [3] 2018	38	M	Adenocarcinoma	IV	DLC + C	24^a^	Penis and pubic	Nodule and ulcer	None	No (2)
Current study 2019										
	45	M	Adenocarcinoma, moderately differentiated	IV	DLC	0^b^	Groin, Perineum	Ulcer	None	No (1)

– = data not reported; AC = adjuvant chemotherapy; ACR = adjuvant chemoradiation; APR = abdominoperineal resection; C = chemotherapy; CR = chemoradiation; F = female; LAR = low anterior resection; M = male; mets = metastasis; NCR = neoadjuvant chemoradiation; NR = neoadjuvant radiation; R = radiation; WLE = wide local excision, DLC = diversion loop colostomy, TPE = total pelvic exenteration, LP = laparoscopic proctectomy, TME = total mesorectal excision, ISR = intersphincteric resection, P = proctectomy, O = oopherctomy.

^c^Described in this article.

a Interval between cancer treatment/diagnosis and skin metastasis presentation.

b In those patients, skin metastasis was the first sign of the underlying malignancy. Therefore, there was no interval between the primary cancer diagnosis and the onset of the skin metastasis.

Advanced-stage disease was found in most of the patients (stage III in 13 patients and stage IV in 15 patients). Surgical intervention, either low anterior resection, diversion, or abdominoperineal resection, was performed, with or without neoadjuvant or adjuvant chemoradiation. 25 patients had recurrent skin metastasis, recurring in less than 2 years in average. In some cases, skin metastasis was the first sign of the underlying malignancy. Skin metastases appeared in several sites, the most common of which was the perineum. Most patients received treatment for skin metastasis in different modalities and vital status was reported for 33 patients. Among these, 23 died.

Only 32 patients reported for follow-up. In average, the period of time between the diagnosis of skin metastasis and death was about 14.8 months (median 7, range 1–60). Multiple cases of isolated cutaneous rectal metastases with no evidence of visceral disease were reported. Also, in a limited number of patients, the cutaneous lesion could occur before the metastasis of any other organ.

Up to date, there is no optimal or standardized strategy with limitation in the management of malignant cutaneous metastasis, but some chemotherapy regimens (including cisplatin, oxaliplatin, irinotecan, capecitabine, and 5-FU) showed good results in control symptoms and increasing survival rate where, for example, combinations of infusional 5FU/LV with irinotecan (FOLFIRI) or FOLFOX helped extend the survival to more than 20 months if compared to surgical intervention [4,28,37].

Also, regimens were reported by Tournigand et al. which prolonged the median survival times, as follows: FOLFIRI followed by FOLFOX to 21.5 months and FOLFOX followed by FOLFIRI to 20.6 months [3,43]. However, multiple cutaneous metastases or unresectable lesions patients can consider systemic chemotherapy. Very limited margins or wide local excision and reconstruction can be considered for isolated skin lesions as recommended in many studies [37,44,45].

## 4 Conclusion

Patient and relatives education, thorough clinical examination during follow-up, and a high index of suspicion are highly recommended with patients who have colorectal cancer or at high risk to develop it. Additional research looking for proper management of such lesions is needed.

## Declaration of Competing Interest

All authors have nothing to disclose.

## Sources of funding

All authors listed below have no source of funding to disclose.

## Ethical approval

There is no ethical approval was obtained as it’s a case report but a written consent was taken from the family as the patient passed away.

## Consent

A written consent was taken from the family as the patient passed away.

## Author contribution

Riyadh Hakami: study concept or design.

Turki Alshammari: study concept or design.

Mohammed alali: data collection, data analysis, interpretation, writing the paper.

Sulaiman AlShammari: data collection, writing the paper.

Zyad Alyahya: data collection.

Mohammed Ayesh: data interpretation (radiology part).

Khaled AlSaad: data analysis interpretation (pathology part).

Alaa Abduljabbar: study concept, design, writing the paper.

## Research studies

Our paper is a case report, no registration was done for it.

## Guarantor

Riyadh Hakami: Drriyadhhakami@gmail.com.

Turki Alshammari: turki.md84@gmail.com.

Alaa Abduljabbar: aabduljabbar@kfshrc.edu.sa.

## Provenance and peer review

Not commissioned, externally peer-reviewed.
